# Big data-driven spatio-temporal heterogeneity analysis of Beijing's catering service industry during the COVID-19 pandemic

**DOI:** 10.1038/s41598-024-51251-z

**Published:** 2024-01-06

**Authors:** Haichao Jia, Minrui Zheng, Peipei Wang, Tianle Li, Xinqi Zheng

**Affiliations:** 1 School of Information Engineering, China University of Geosciences, Beijing, 100083 China; 2 School of Public Administration and Policy, Renmin University of China, Beijing, 100872 China; 3 Digital Government and National Governance Lab, Renmin University of China, Beijing, 100872 China; 4 Technology Innovation Center for Territory Spatial Big-Data, MNR of China, Beijing, 100036 China; 5 Beijing Fangshan Observation and Research Station of Comprehensive Exploration Technology, Ministry of Natural Resources of People’s Republic of China, Beijing, 102400 China

**Keywords:** Socioeconomic scenarios, Sustainability, Health care economics

## Abstract

The Catering Service Industry (CSI) experienced profound impacts due to the COVID-19 pandemic. However, the long-term and multi-timepoint analysis using big data remained limited, influencing governmental decision-making. We applied Kernel Density Estimation, Shannon Diversity Index, and the Geographic detector to explore the spatial heterogeneity and determinants of the CSI in Beijing during the pandemic, with monthly granularity. The temporal-spatial dynamics of the CSI presented a "W"-shaped trend from 2018 to 2023, with pivotal shifts aligning with key pandemic stages. Spatial characteristics exhibited heterogeneity, with greater stability in the city center and more pronounced shifts in peripheral urban zones. Districts facing intricate outbreaks showed lower catering income, and Chinese eateries exhibited heightened resilience compared to others. The CSI displayed strong interconnections with living service sectors. Development in each district was influenced by economic level, population distribution, service facilities convenience, and the risk of the COVID-19 pandemic. Dominant factors included total retail sales of consumer goods, permanent population, average Baidu Heat Index, density of transportation and catering service facilities, infection cases and the consecutive days with confirmed cases existing. Consequently, we suggested seizing post-pandemic recovery as an avenue to unlock the CSI's substantial potential, ushering a fresh phase of growth.

## Introduction

The COVID-19 epidemic spread dramatically throughout 2020 and became a global pandemic^1^. To contain the extensive and rapid spread of COVID-19, numerous countries enacted stringent measures, such as social distancing, prohibition of gatherings and closure of public transit^2–4^. Despite suitable policy responses having effectively controlled the widespread transmission of the pandemic, they inflicted devastating impacts on numerous industries, with the Catering Service Industry (CSI) bearing the brunt of the impact^5,6^. The CSI heavily relies on mobility and gatherings, but it experienced a decline in consumer spending on dining out due to mobility restrictions and lockdown policies^7^. Moreover, the COVID-19 pandemic brought about significant changes in consumer behavior^8^. People increasingly opted for contactless alternatives from restaurants, such as online ordering and delivery services, while avoiding dine-in services^9^. This transformation undoubtedly led to increased operational costs and introduced new competitive dynamics, placing substantial pressure on the survival and operations of the CSI^10^. However, as the pandemic situation evolved, the prevention and control policies adjusted. In addition, the government implemented a series of robust support measures^11^, such as subsidies, debt deferment, and facilitation of financing, which facilitated the recovery of the CSI. The CSI stands out as a vital life service industry, capable of reflecting the local socio-economic attributes, the intensity of local economic activities, and the imbalance in economic development^12^. Against the backdrop of the COVID-19 pandemic, examining CSI fluctuations becomes a valuable tool to capture changes in local societies and economies^13^. This is particularly crucial for assessing the specific impacts of the pandemic on human life. Therefore, the industry trends of the CSI during the COVID-19 pandemic could serve as a reflection of multifaceted information, including government policies, pandemic dynamics, and economic performance. For the temporal and spatial evolution of the CSI, long-term monitoring at various scales provides targeted information for assessing the impact of the pandemic and guiding industry development. Therefore, utilizing extensive data support, a meticulous analysis of the spatiotemporal characteristics of the CSI during the COVID-19 pandemic holds significant importance. Such analysis contributes to the establishment of risk mitigation strategies for the CSI, informs future planning by relevant authorities, and plays a pivotal role in driving urban economic development.

With the increasing integration of spatiotemporal data and mathematical models, spatiotemporal analysis has gained significant traction in COVID-19 pandemic data research, encompassing aspects such as analysis^14,15^, prediction^16,17^, and influencing factors^18,19^. Furthermore, research on the social^20,21^, environmental^22,23^, and economic aspects^24,25^ of the pandemic has deepened in response to public health crises. Current research on the CSI during the pandemic predominantly relies on qualitative surveys or limited spatiotemporal coverage data to analyze its spatial patterns and associated impacts. Nevertheless, conducting long-term, multi-timepoint analyses using big data could potentially facilitate more in-depth exploration^26^. Firstly, existing research based on multiple data sources like points of interest (POI) rarely engages long-term dynamic monitoring and analysis of specific spatial areas^27^. Most of them conduct static analysis at a particular point in time, lacking temporal continuity. Wang et al. employed a GIS-based analytical framework, constructed a dataset including POI (2020), trajectory and population data to evaluate the impact of COVID-19 on the CSI^28^. Secondly, there is a need for more refinement in the monitoring time scale of data. They often at an annual time scale, and are unable to capture the exact monthly granularity of POI data collection. Deng et al. scrutinized the changes in CSI facilities in Wuhan before and after the COVID-19 pandemic using POI in 2019 and 2021^29^. Finally, there is an imbalance in research related to the impact of unexpected events, with a lack of monitoring and analysis at critical time points during the pandemic^28^. Existing research often combines big data for macro-level analysis, primarily focusing on spatial overall layout characteristics, with limited content focusing on micro level and hierarchical research^30^. For example, horizontal comparisons of different types of catering spaces and the differences in various types of catering spaces under the influence of the pandemic require further research^31^. Feizizadeh et al. obtained the location and orders of restaurants from Tabriz city in 2020, factoring in population and traffic characteristics. They employed integrated GIS decision rules to analyze the level of food delivery services in cities during the COVID-19 pandemic^32^. But they did not segment different types of restaurants and consider the impact of restaurant attributes on the analysis results.

We acknowledged the importance of CSI in the social economy and its prominent impact during the COVID-19 epidemic. This paper used Beijing as an example to further study the spatial characteristics evolution and influencing factors of CSI under spatiotemporal differences. To analyze the fine-grained and continuous spatiotemporal patterns of the CSI, we considered the overall characteristics and individual differences of the CSI, as well as the quantity and quality of spatial distribution, from the perspectives of time and space. This paper utilized data from various time points, ranging from 2018 to 2023 in Beijing, including POI data, spatial vector data, and statistical data. We employed Kernel Density Estimation (KDE), Standard Deviation Ellipse (SDE), Shannon Diversity Index (SHDI) and Geographic detector to analyze the changes in the quantity, spatial clustering, and functional diversity of catering service facilities in Beijing. We refined the categories of catering service facilities and examined the differences in characteristic changes of different types of catering spaces. Through these methods, we explored the dynamic evolution of the spatiotemporal characteristics of the CSI in Beijing during the pandemic, considering the various stages of the pandemic over the past few years, especially during 2022. We discovered the spatial heterogeneity of CSI in Beijing during the pandemic and further explored the factors that influencing the development of CSI. Moreover, this case study in Beijing serves to facilitate further investigations into the impact of current urban policies. It provides guidance for urban policymakers in managing post-pandemic development and offers developmental insights for various industries, including the CSI.

## Methodology

### Study region

**Figure 1:**
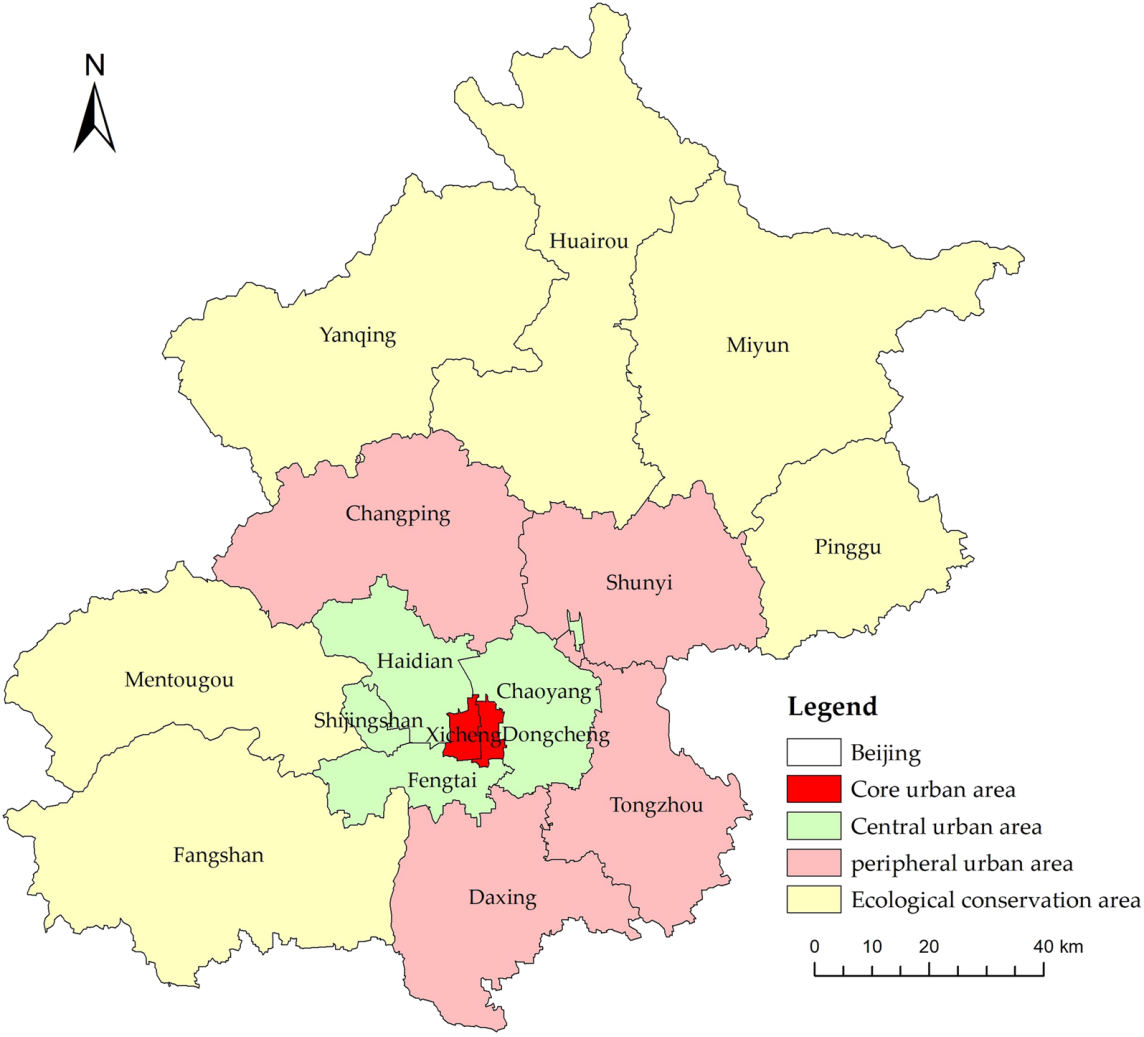
Schematic division of four types of urban areas in Beijing, referencing the Spatial Structure Planning Map of Beijing Municipality. The figure was drawn with ArcGIS software version 10.7 (https://www.arcgis.com/index.html).

Beijing (115.7°–117.4°E, 39.4°–41.6°N) spans an approximate area of 16,400 km^2^ and is composed of 16 administrative districts. It is also China's political, cultural, international exchange and technological innovation center. Over the years, Beijing has been strategically evolving towards a path emphasizing livability and high quality. Due to its substantial size, distinguished status, and evolving developmental approach, Beijing was selected as the primary focus area for this study. In accordance with the *Beijing Urban Master Plan (2016–2035)*, we categorized these sixteen districts into four distinct urban classifications for comprehensive analysis, as depicted in Fig. 1. The core urban areas consisted of Dongcheng and Xicheng districts, as the central hub for the capital's functions. The central urban areas encompassed Chaoyang, Fengtai, Haidian, Shijingshan districts. These districts play a crucial role in the construction of an internationally top-tier, harmonious, and livable capital city, and they are the primary areas for relocating non-capital functions. The peripheral urban areas included Tongzhou, Shunyi, Daxing and Changping districts. They are key regions for accommodating suitable functions and population dispersal from the central urban area. The ecological conservation areas comprised Mentougou, Pinggu, Huairou, Miyun, Yanqing and Fangshan districts, playing a vital role in securing the sustainable development of the capital.

### Data sources

The POI data of Beijing was downloaded through the API interface and POI classification standards provided by Amap (https://console.amap.com/). We collected POI data from 2018 to 2023, including latitude, longitude, category, address, and name. The POI dataset underwent rigorous data cleansing, which involved deduplication and error correction to enhance accuracy. We precisely recorded the data collection time down to the year and month, and the dataset included 857,814 entries of catering-related POI in total, as shown in Table 1.

**Table 1:** Basic information of catering-related POI data in Beijing.

Datetime	2018.07	2019.06	2020.11	2021.12	2022.05	2022.06	2022.10	2023.01	Total
pcs	93,508	103,045	98,751	118,639	98,199	110,722	118,220	116,730	857,814

This study pertained to historical data on Beijing's catering-related POI. Ideally, the POI historical database should be fixed intervals and cross-referenced with the environmental context at the time, gradually forming a cumulative historical database. To validate location accuracy, we adopted a comprehensive approach, involving a comparative analysis of POI counts and content with alternative data sources. Additionally, we conducted keyword searches using Python through Amap's API, such as dessert shop. We compared the retrieved POI data in Beijing's Haidian district with the data in our database. The results indicated a high level of precision in the POI data. Employing Amap's POI classification standards (mid-level classification), we reclassified the POI data into six categories: "Catering-Related Establishments, Leisure Dining Places, Chinese Restaurants, Foreign Restaurants, Fast Food Restaurants, and Beverage and Dessert Establishments." This reclassification facilitated the subsequent analysis of the diversity of catering services in Beijing.

Currently, the collection of POI data depends on online maps. The efficiency of data updates is influenced by various factors, making it challenging to accurately estimate the speed of data refresh. This may involve the issue of the data "lag". We selected the POI data from various time points to enable a more nuanced temporal analysis. The collection times for these datasets were specifically aligned with crucial phases in the pandemic's evolution, all while maintaining a certain time interval. This methodology potentially allows for a buffer period for the timely updating of POI data. In this study, the most recent interval between POI datasets was one month, precisely covering the period from May to June 2022. Both datasets exhibited certain divergences, indicating, to a certain extent, the updates in POI data during that specific month.

The data on COVID-19 infection cases, prevention measures, and related information in Beijing were collected from official websites. These websites included the National Government Service Platform (http://gjzwfw.www.gov.cn/), the National Health Commission (http://www.nhc.gov.cn/), and the Beijing Municipal Center for Disease Control and Prevention (https://www.bjcdc.org/). We obtained dynamic information regarding the COVID-19 pandemic situation in Beijing, including the distribution of COVID-19 cases in the first half of 2022.

The GDP and population data for each district were obtained from the Beijing Municipal Bureau of Statistics (https://nj.tjj.beijing.gov.cn/) and respective district government official platforms. We compiled the resident population data for various districts in Beijing (measured in individuals) from the Seventh National Population Census. Additionally, this study collected the overall economic performance data of Beijing's districts during different phases from 2018 to 2023, including catering revenue, total retail sales of consumer goods, and per capita disposable income.

The Baidu Heat Map (BHM) was sourced from Baidu Maps. Leveraging the Baidu Maps API endpoint, we gathered population heat data for Beijing on May 28, 2022, at 12:00. The heat map underwent vectorization for visualizing population data. Subsequently, we organized and computed the average Baidu Heat Index (BHI) for each district in Beijing, serving as indicators of population vitality and mobility across the city.

### Dynamic status of COVID-19

In response to the COVID-19 pandemic, Beijing initiated a Level One public health emergency response on January 24, 2020. Subsequently, the city established and refined a normalized epidemic prevention and control mechanism, navigating a prolonged landscape marked by localized cluster outbreaks, sporadic cases, and dynamic adjustments to risk levels and control strategies. This intricate situation continued until the close of 2022. Noteworthy large-scale cluster outbreaks encompassed the outbreak in the Xinfadi market in June 2020 and the outbreak involving the Tiantang supermarket and bar in the first half of 2022. This study conducted an intricate investigation based on the COVID-19 epidemic data of 2022.

**Figure 2:**
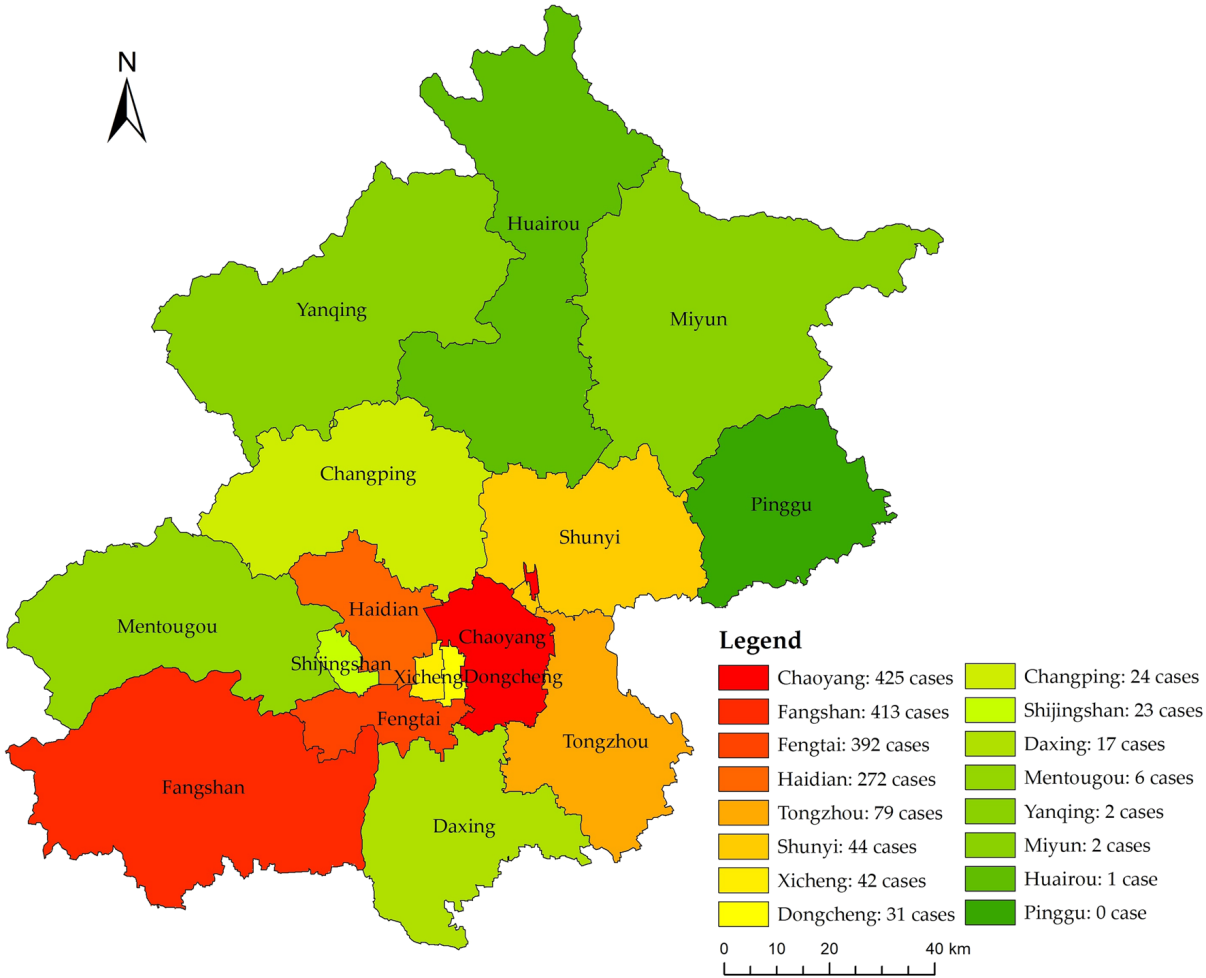
Distribution of COVID-19 infection cases in Beijing from April 22, 2022, to June 1, 2022. The figure was drawn with ArcGIS software version 10.7 (https://www.arcgis.com/index.html).

Between April 22 and June 2022, Beijing experienced a significant cluster outbreak associated with the Tiantang supermarket and bar. The challenges in disease prevention and control surpassed those encountered during the cluster outbreak at the Xinfadi market in 2020. The total confirmed cases exceeded one thousand. The distribution of infections from April 22 to June 1 is depicted in Fig. 2. This outbreak spanned across 15 districts, with elevated and moderate risk zones concentrated in Chaoyang, Fangshan, Fengtai, Haidian, and Tongzhou districts. To effectively mitigate the spread of the outbreak, Beijing promptly implemented stringent control measures, including the temporary suspension of in-person dining services and the mandate of a negative nucleic acid test result within 48 h as a prerequisite for accessing public venues.

### Research method

#### Kernel Density Estimation

The method calculates the density of features within their surrounding neighborhoods, reflecting the spatial distribution characteristics of these features^33^. The higher kernel density value indicates a denser spatial distribution of catering-related POI^34^. This study utilized the technique to analyze the clustering characteristics of catering service facilities in Beijing. To emphasize significant differences between various years, breakpoints for kernel density values were manually set, excluding the maximum and minimum values. A consistent color scheme was used to represent similar ranges of kernel density values, facilitating the analysis and comparison of data distribution areas and patterns for each year.

1 $$\hat{f}(x) = \frac{1}{nh}\sum\limits_{i = 1}^{n} {k\left( {\frac{{x - x_{i} }}{h}} \right)}$$

where $\hat{f}(x)$ stands for the estimated kernel density value at spatial location $x$, $h$ is the distance attenuation threshold (bandwidth), $n$ signifies the observed count of catering-related POI, $k$ function represents the spatial weight function, $x - x_{i}$ represents the distance between the estimation point $x$ and the sample observation point $x_{i}$.

#### Standard Deviation Ellipse

The method reveals the spatial distribution direction in the region according to the density, orientation and quantity of points, and assesses the concentration degree of its distribution^35^. The paper used it to examine the variations in the spatial distribution of Beijing's CSI at a monthly granularity during the COVID-19 pandemic.

2 $$E_{x} = \sqrt {\frac{{\sum\nolimits_{i}^{n} {\left( {x_{i} - \overline{X}} \right)} }}{n}}$$

3 $$E_{y} = \sqrt {\frac{{\sum\nolimits_{i}^{n} {\left( {y_{i} - \overline{Y}} \right)} }}{n}}$$

where $E_{x}$ and $E_{y}$ represent the lengths of the major and minor axes of the standard deviation ellipse, $n$ is the number of catering-related POI, $(x_{i} ,y_{i} )$ denotes the spatial location of the $i$-th catering-related POI, $(\overline{X},\overline{Y})$ is the average center of all catering-related POI.

#### Shannon Diversity Index

The method characterizes the complexity and variability of different patch types within a landscape^36^. A higher value is indicative of greater patch richness or a more even distribution of patch areas^37^. Based on the POI reclassification results, this paper calculated the SHDI values of Beijing's CSI at various locations. This computation aimed to depict the variations in the functional diversity of the CSI within Beijing. A higher SHDI value signifies greater diversity in the distribution of catering facilities for a given year and area.

4 $$H = - \sum\limits_{i = 1}^{S} {P_{i} } \ln P_{i}$$

where $S$ represents the count of catering-related POI types in the sample, $P_{i} = \frac{{N_{i} }}{N}$, $N_{i}$ is the number of the $i$-th POI type, $N$ denotes the sum of all POI types in the sample.

#### Geographic detector

This is an analytical model utilized to uncover the spatial differentiation of a certain geographical attribute and the driving forces underpinning it^38^. It is widely applied in studying the influencing factors of various events such as natural phenomena, social dynamics, and human health^39^. In this research, the factor detection module of the Geographic detector was employed to investigate the driving factors behind the spatial heterogeneity of Beijing's CSI across different districts during the COVID-19 pandemic. Additionally, the study examined the explanatory capacity of each individual factor.

5 $$q_{X} = 1 - \frac{1}{{N\sigma^{2} }} \sum\limits_{h = 1}^{L} {N_{h} \sigma_{h}^{2} }$$

where $q_{X}$ is the explanatory power value of the detection factor $X$, with a range of [0,1]. A higher value indicates a stronger explanatory degree of the influencing factor $X$ on $Y$, while a lower value implies a weaker relationship^40^. $L$ represents variable $Y$ or factor $X$ stratification, manifesting as categorization or zoning. $N_{h}$ and $N$ stand respectively for the number of units in layer ${\text{h}}$ and the entire area. $\sigma_{h}^{2}$ and $\sigma^{2}$ denote the variances of the $Y$ values for layer ${\text{h}}$ and the entire region correspondingly.

#### Selection of detection factors

As a pivotal industry for boosting domestic demand, promoting consumption, stabilizing growth, and benefiting people's livelihoods, the CSI exhibits a spatial distribution pattern influenced by multifaceted factors across various scales. The research indicated that the spatial distribution and development of the CSI were influenced by factors such as regional economic level, population distribution, and the accessibility of service facilities^41^. According to the *China Catering Industry Development Report* from 2020 to 2022, the CSI experienced the impact of the COVID-19 pandemic. Given the context of the pandemic, we considered the potential influence of COVID-19 pandemic risk on the CSI. Building upon existing theories and research, this paper explored the influencing factors of the CSI's development in various districts of Beijing, focusing on economic level, population distribution, the convenience of service facilities, and the risk of the COVID-19 pandemic.

The study focused on the 16 districts of Beijing as its research subjects. We utilized COVID-19-related data from April 22, 2022, to June 1, 2022, along with socio-economic data. Catering revenue serves as a proxy for the development level of the CSI to some extent, providing a straightforward representation of data. High or consistently increasing catering revenue indicates a robust development in the catering market. The dependent variable (Y) was chosen as the catering revenue for each district. Across the dimensions of economic level, population distribution, the convenience of service facilities, and the risk of the COVID-19 pandemic, eight influencing factors were selected. These factors were depicted in Table 2.

**Table 2:** Detection factors of spatiotemporal changes of CSI in Beijing.

Driving mechanism	Detection factors	Code	Grade
economic level	Total retail sales of consumer goods	X1	3
	Per capita disposable income	X2	3
population distribution	Population density	X3	3
	Average Baidu Heat Index	X4	3
the convenience of service facilities	Density of transportation facilities	X5	3
	Density of catering service facilities	X6	3
the risk of the COVID-19 pandemic	Tally of infection cases	X7	3
	The consecutive days with confirmed cases existing	X8	3

If a city district reported new COVID-19 infections on a given day, that day was designated as "a day with confirmed cases existing." X8 represented the cumulative total of these days.

1. *Economic level* Higher economic level fosters the amplification of agglomerative effects of diverse production factors, propelling swift growth across sectors, including the CSI^42^. Areas with elevated economic development levels showcase intensified economic activities, where human flows, logistics, and information converge, leading to higher catering service density^12^. The gross regional product (GRP) stands as the paramount indicator for reflecting economic conditions, but it is challenging to obtain. This is due to incomplete and varying presentations of economic data across different districts in Beijing. According to the official explanation from the *National Bureau of Statistics of China*, the total retail sales of social consumer goods stand as a crucial indicator reflecting the overall retail market, including the CSI, and the general economic well-being. We selected the total retail sales of social consumer goods as an indicator aimed to reflect the regional economic level. Regional economic conditions exert influence over residents' purchasing power, directly impacting merchants' sales profits, thus affecting the outreach and viability of the CSI. Subsequently, per capita disposable income was chosen as the second indicator to portray the regional economic status^12^.
2. *Population distribution* The CSI, being a part of the life service sector, heavily relies on the local population^13^. Consumer demand for catering plays a pivotal role in determining the industry's sustainability. A rise in population and increased vitality contribute to the flourishing development of the catering service sector within a region^43^. Permanent population serves as a direct indicator of population distribution across various districts in Beijing. However, due to the dynamic nature of population mobility, this factor requires consideration. Baidu Heat Index (BHI), extracted from Baidu Heat Map (BHM), offers insights into the volume of people movement in each district. BHM, utilizing geographical location data to portray human activities, is extensively utilized for assessing people's mobility in urban spaces or the vibrancy of distinct regions^44^. We selected the heatmap from noon on May 28, 2022, representing the later stage of the recent wave of the pandemic and ensuring a certain level of data representativeness.
3. *The convenience of service facilities* Considering the service features of the CSI, we selected the convenience of transportation and the abundance of catering service facilities to represent service facility accessibility. The convenience of transportation reflects the accessibility of restaurants, with higher accessibility indicating greater potential customer demand. The density of transportation facilities is a pivotal indicator of accessibility^45^. A richer array of catering service facilities can offer consumers more dining options, providing a broader range of services^43^. The density of catering service facilities can reflect the abundance of catering service amenities.
4. *The risk of the COVID-19 pandemic* In response to the COVID-19 pandemic, measures aimed at restraining virus spread and reducing gatherings, including home quarantine and the temporary suspension of dine-in services, impacted the daily operations of the CSI^32^. The heightened operational costs, additional expenditures on delivery commissions, and changes in consumer behavior collectively created challenging circumstances for the CSI^46^. The risk of the COVID-19 pandemic was visually reflected in the number of infections^47^. As infections increased, the risk level and control measures in a district were intensified. Control policies in a district remained in place until the department reported "zero infections" for consecutive days, signaling the easing of control measures. Therefore, the more days a district reported the presence of confirmed cases, the more it indirectly indicated the district's higher risk of facing the COVID-19 pandemic.

## Results

### Spatio-temporal evolution of CSI

#### Fluctuations of catering-related POI quantity

**Figure 3:**
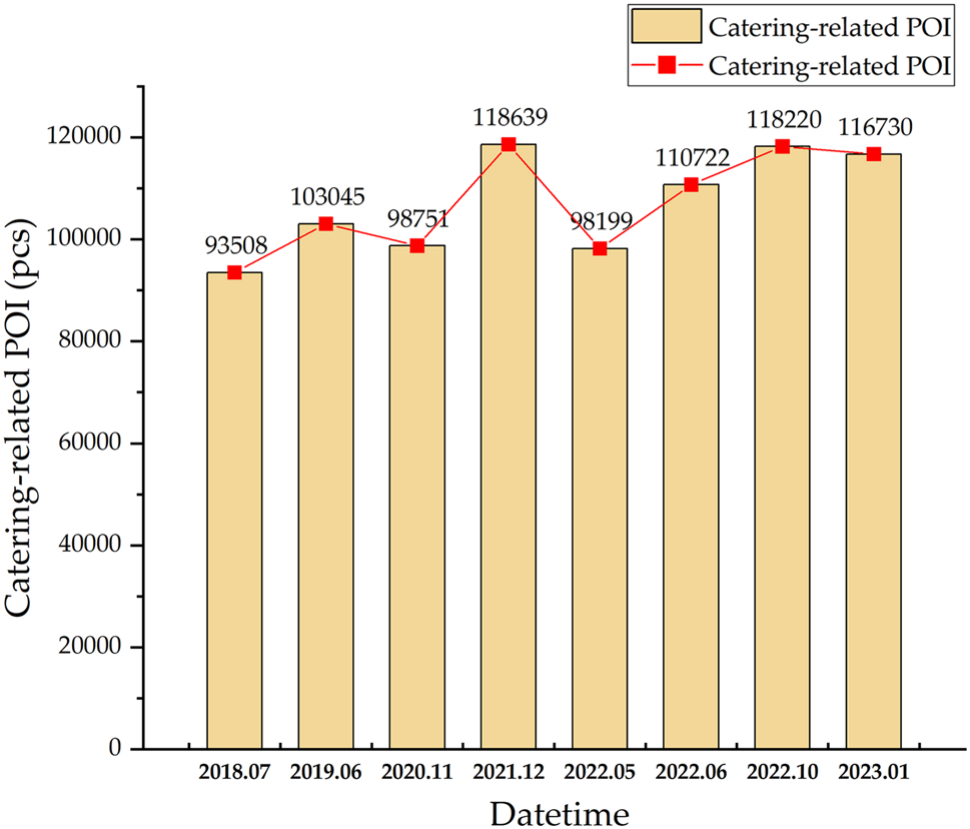
Variations in the quantity of catering-related POI in Beijing. The figure was drawn with Origin software version 2023 (https://www.originlab.com/).

We conducted statistical analysis on the fluctuations in the quantity of catering-related POI. The fluctuations in the quantity of catering-related POI in Beijing from 2018 to 2023 corresponded closely to the dynamics of the COVID-19 pandemic. This pattern exhibited an overall "W" shaped fluctuation, with pivotal shifts occurring in November 2020, December 2021, and May 2022, as depicted in Fig. 3. According to data from the China Cuisine Association, the total revenue of the national CSI in the first quarter of 2020 was only 602.6 billion yuan, representing a year-on-year decline of 44.3%. From March, CSI gradually resumed across various regions, and the market began recovering. By November 2020, the total annual revenue of Chinese CSI reached 3.46 trillion yuan, showing an 18.6% year-on-year decrease. Considering the national context of the CSI and the evolving COVID-19 situation in Beijing, although the CSI in Beijing had entered a recovery phase, the pace of recovery was modest. The number of POI records remained relatively low, only slightly surpassing the data observed in May 2022, when there was a significant outbreak. Prior to the outbreak of the pandemic in 2018 and 2019, the number of POI was on an upward trajectory, but Beijing broke this trend in 2020. In 2021, as the pandemic stabilized in various regions and due to various policy supports, the number of POI increased rapidly, contributing to a significant recovery of the CSI. In the first half of 2022, Beijing experienced several localized outbreaks of the pandemic, causing a setback in the CSI. The number of catering-related POI in May 2022 was notably lower than in 2021. From June 2022 to January 2023, with changes in the pandemic situation and control policies, there was a fluctuating increase in the number of POI.

We computed the change rate in the number of catering-related POI in various urban districts of Beijing over different years. Combining this with Beijing's urban spatial structure planning (categorized into four types of urban districts), the results revealed a correlation between the rate of change in the number of catering-related POIs in different urban districts and their respective categories. During 2020 and 2021, urban areas with a higher rate of change in POI numbers were predominantly in ecological conservation zones, while more stable trends were observed in core and central districts. The maximum negative growth rate occurred in November 2020, observed in Yanqing District at − 44.61%. A total of nine urban areas exhibited negative growth rates in 2020, with all six areas within the ecological conservation zone showing substantial negative growth, above − 20%. The maximum positive growth rate was observed in 2021, at 126.99% in the Mentougou district. Areas with significantly positive growth rates were still concentrated throughout the entire ecological conservation region, ranging from 68.92 to 126.99%. Notably, in May 2022, urban areas with a higher number of confirmed cases experienced prominent negative growth in POI numbers. For instance, Fengtai, Chaoyang, and Fangshan districts all demonstrated negative growth rates of around − 20%. Dongcheng and Xicheng districts exhibited the most substantial negative growth rates, recorded at − 23.30% and − 22.90% respectively. From the perspective of Beijing's urban district characteristics, the stability and resilience of the CSI in peripheral urban districts of Beijing appeared relatively fragile, while more developed urban districts closer to the city center exhibited relative stability.

#### Changes of CSI agglomeration level

**Figure 4:**
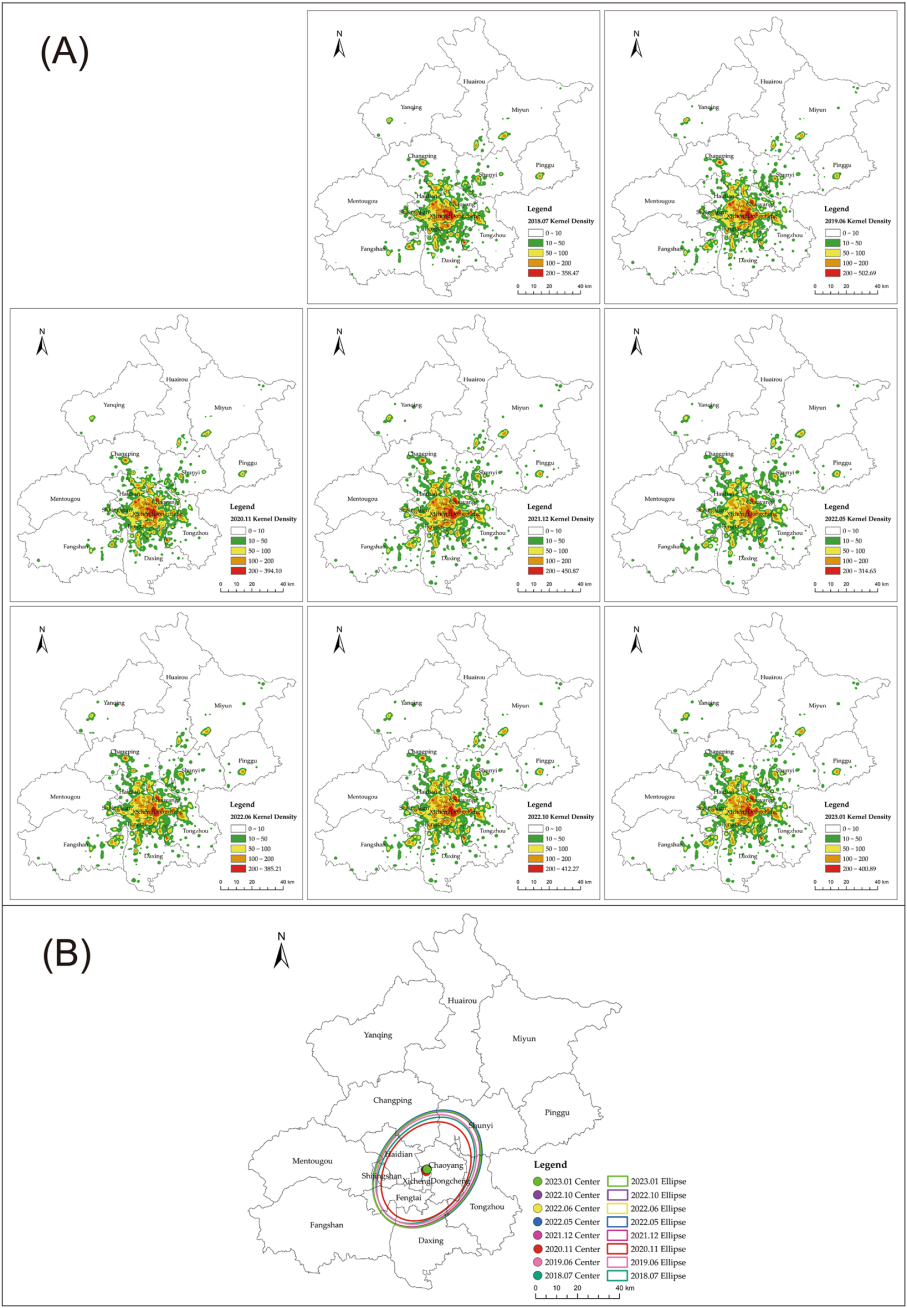
(**A**) Kernel density of catering-related POI in Beijing from 2018 to 2023. (**B**) Distribution of gravity center and standard deviational ellipse of the CSI in Beijing from 2018 to 2023. The figure was drawn with ArcGIS software version 10.7 (https://www.arcgis.com/index.html).

To further explore the spatial characteristics of the CSI during the COVID-19 pandemic in Beijing, we applied the KDE and SDE to analyze the heterogeneity of the agglomeration characteristics of the CSI based on the annual catering-related POI data. The spatiotemporal variations in the agglomeration characteristics of the CSI in Beijing from 2018 to 2023 exhibited similarities with the number of catering-related POI fluctuations. The degree of agglomeration varied in response to the development of the pandemic, continuing to follow a "W"-shaped fluctuation trend, as illustrated in Fig. 4A. Around significant pandemic events, specifically in 2020 and May 2022, the degree of agglomeration decreased. Conversely, during stable pandemic conditions, such as in October 2021 and October 2022, the degree of agglomeration deepened.

The spatial distribution of CSI in Beijing demonstrated a pronounced "one core, multiple centers" configuration. The degree of agglomeration in the CSI gradually decreased from the core urban area to the central urban area, further to the peripheral urban area, and eventually to the ecological conservation area. The zenith of agglomeration was concentrated in the western precinct of Chaoyang district and the eastern sector of Dongcheng district, adhering to a distribution model typified by "centralized concentration and peripheral diffusion." For the entire city of Beijing, in November 2020, the degree of agglomeration within the CSI registered a significant decline compared to the preceding and ensuing years. The city's highest kernel density value for the entire city amounted to 394.10 in 2020, which was lower than the figures of 502.69 in 2019 and 450.87 in 2021. Similarly, in May 2022, the extent of agglomeration in the CSI was lower when juxtaposed with both June and October of the same year. Throughout both November 2020 and May 2022, the two prominent time points of change, the agglomeration dynamics of the CSI retained their uniformity. High-level agglomeration areas were reduced or downgraded, particularly in the core and central urban areas, while low-level agglomeration points disappeared, with a notable presence in the ecological conservation areas.

We employed standard deviation ellipses for further analysis of Beijing's CSI spatial layout, as illustrated in Fig. 4B. From 2018 to 2023, the standard deviation ellipses exhibited a southward inclination over the entire urban area, following a northeast-to-southwest orientation. The directional alignment remained consistent across these years, with minimal deviation of the ellipse center. Considering the extent of the standard deviation ellipses, in November 2020, the standard deviation ellipse for catering-related POI exhibited the shortest perimeter at 141.18 km and the smallest area at 1538.62 km^2^. This indicated that the distribution range of the CSI in Beijing contracted significantly in 2020, reflecting constrained development during that year. Following December 2021, the scope of the standard deviation ellipses expanded, with areas exceeding 2000 km^2^, surpassing the ellipse areas of both 2018 and 2019. This signified a broader spatial distribution of CSI in Beijing compared to the pre-pandemic.

#### Variation of catering facility diversity

**Figure 5:**
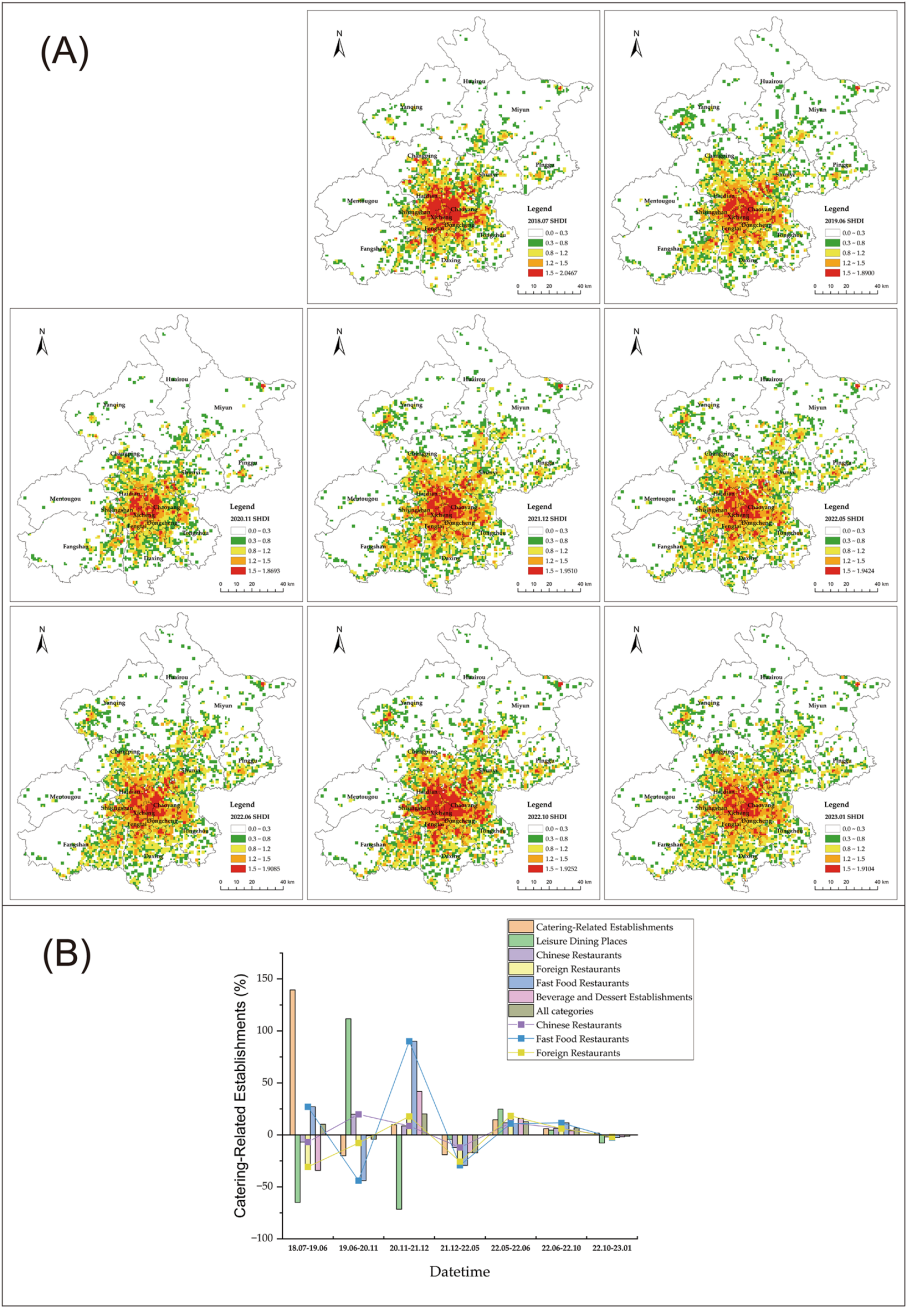
(**A**) Multi-function mix diversity of Beijing's CSI from 2018 to 2023. (**B**) Variation rates of different types of catering service facilities in Beijing. The x-axis represents time spans for comparing the quantities of different types of catering service facilities. For instance, "18.07–19.06" indicates the change in the quantity of a specific type of catering service facility in June 2019 compared to July 2018. The y-axis represents the change rate of the quantities of these facilities. The figure (**A**) was drawn with ArcGIS software version 10.7 (https://www.arcgis.com/index.html). The figure (**B**) was plotted with Origin software version 2023 (https://www.originlab.com/).

In the comprehensive analysis of CSI in Beijing, it was observed that spatiotemporal heterogeneity existed in both the quantity distribution and degree of agglomeration. Furthermore, variations in spatiotemporal patterns were also identified among different types of catering facilities (Fig. 5A). We calculated the SHDI for various catering service facilities in each district of Beijing from 2018 to 2023. The SHDI was employed to gauge the functional diversity of catering service facilities in Beijing. Generally, the SHDI values across the city exhibited a spatial pattern of "high in the center and low in the surroundings". The spatial distribution quality of catering service facilities was notably uneven, with high SHDI values predominantly concentrated in pixels within the core and central urban districts, while lower SHDI values were prevalent in pixels situated in the peripheral areas of Beijing. This pattern was consistent with the agglomeration characteristics of the overall CSI. The most prominent fluctuation in SHDI values in Beijing occurred in 2020. Numerous areas within the core and central urban regions experienced a significant decrease in high SHDI values, transitioning to moderate and low-value zones. Within the peripheral urban and ecological conservation zones, a substantial number of mid-level SHDI pixel values transformed into low-value regions. Commencing in 2021, SHDI values in various districts exhibited an upward trend within dynamic fluctuations, mitigating the previously observed decline. Across different regions, the diversity of catering services available to residents improved to varying degrees, a trend primarily driven by the recovery of Beijing's CSI.

We analyzed the rate of change in the quantity of six categories of CSI establishments in Beijing, as shown in Fig. 5B. The number of Chinese restaurants remained relatively stable overall, and even experienced a slight increase in 2020 with a growth rate of 19.82%. Despite fluctuations in the quantity of various types of establishments in May and June 2022, Chinese restaurants exhibited comparatively lower rates of change, at − 12.13% and 11.63%, respectively. Chinese restaurants ranked fifth when ranking the rate of change in quantity for the six categories from highest to lowest. This positioning suggested that Chinese restaurants displayed a certain level of stability and resilience in the context of the pandemic. In 2020, the category with the largest negative growth in quantity was fast-food restaurants, with a negative growth rate of − 43.91%. Conversely, in 2021, fast-food restaurants had the highest positive growth rate, at 90.09%. Similarly, in May and June 2022, fast-food restaurants experienced significant fluctuations in quantity. This suggested that fast-food restaurants exhibited relatively low stability but possess a degree of resilience. Foreign restaurants also displayed characteristics similar to fast-food restaurants. Leisure dining establishments, which include flexible attributes such as fresh fruit bars and coffee restaurants, exhibited a prominent and irregular rate of change in quantity, with no clear pattern discernible.

### Driving forces of CSI evolvement

#### Strong interconnections among living services

The CSI falls within the realm of the life service industry, intimately intertwined with the fabric of human existence. As diverse life service sectors rarely operate in isolation, it becomes crucial to examine the spatial correlations among these different services. We utilized correlation coefficients (R) to investigate the linear relationships among variables. When ∣R∣ < 0.4, it indicates a weak correlation; 0.4 < ∣R∣ < 0.6, it suggests a moderate correlation; 0.6 < ∣R∣ < 0.8, it indicates a strong correlation; ∣R∣ > 0.8, it denotes a very strong correlation. After standardizing the POI density for diverse facilities and computing correlation coefficients, we conducted a spatial correlation analysis on the density among the seven distinct types of life service industries in Beijing. The results consistently revealed patterns across different years, with some correlation coefficients displaying a maximum numerical difference of 0.05. Ultimately, we focused on the POI data from the year 2020, a pivotal period in the development of the COVID-19 pandemic, to enable an analysis within the context of the pandemic.

**Figure 6:**
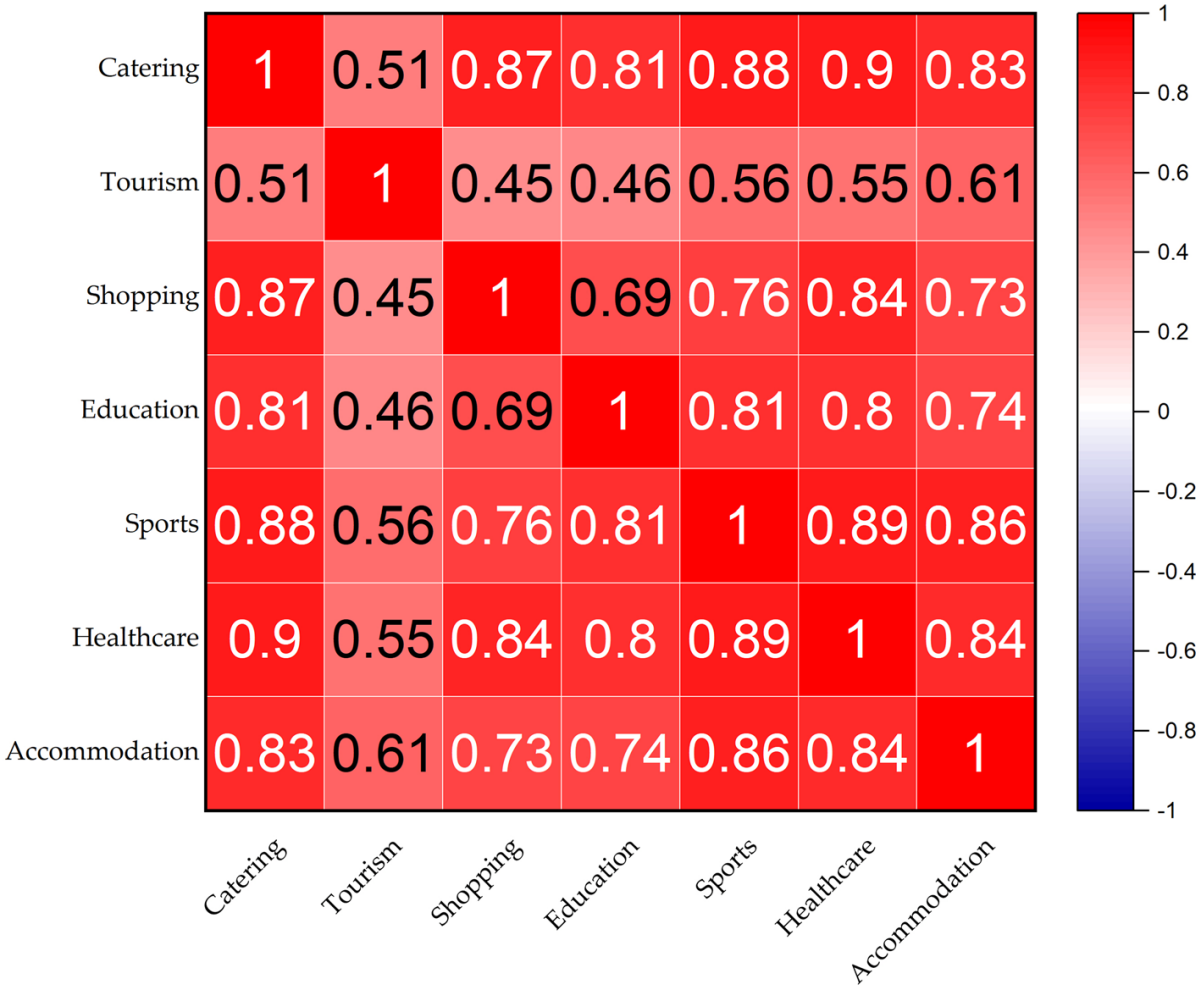
Spatial correlation of various service sectors in Beijing. The results were derived from the data of the year 2020. The figure was plotted with Origin software version 2023 (https://www.originlab.com/).

The spatial distribution of various urban living service sectors in Beijing exhibits significant correlations (Fig. 6). The R between the CSI and various living service sectors exceeded 0.8, indicating a robust correlation. The R between the CSI and scenic spots was 0.51, reflecting a moderate correlation between the CSI and the tourism sector. The CSI serves as a foundational component of urban living services, and each type of service facility within this sector relies significantly on it. The development of various urban living service sectors is intrinsically linked to the support provided by the CSI. Amidst the backdrop of the COVID-19 pandemic, statistics from the Beijing Municipal Bureau of Statistics revealed that the entire service-oriented consumer market in the city faced disruptions. Various urban living service sectors experienced slowdowns or even regression in their development, leading to mutual influences and constraints.

#### Influencing factors and mechanisms

To delve into the determinants of the development of the CSI in Beijing, we initially standardized all the indicators. Subsequently, these standardized influencing factors were imported into the Geographic detector to calculate their explanatory power for the revenue of the CSI in Beijing (Table 3). All eight factors demonstrated an impact on the development of the CSI. Except for per capita disposable income, the remaining factors were statistically significant at the 0.05 level, with explanatory power ranging from 50 to 80%. The spatiotemporal pattern of Beijing's CSI was collectively shaped by factors such as economic level, population distribution, the convenience of service facilities, and the risk of the COVID-19 pandemic. The dominant driving factors included total retail sales of consumer goods, permanent population, average BHI, density of transportation facilities, density of catering service facilities, tally of infection cases, and the consecutive days with confirmed cases.

**Table 3:** Geographic detector results of influencing factors of the CSI spatio-temporal variation in Beijing.

Detection factors	X1	X2	X3	X4	X5	X6	X7	X8
q	0.7672	0.3647	0.6573	0.5877	0.5877	0.5877	0.5585	0.5902
p	0.0074	0.1331	0.0459	0.0344	0.0344	0.0344	0.0403	0.0273

Economic level stands as a pivotal and dynamic facet of regional infrastructure development. Generally, regions with elevated economic status boast significant advantages across resources, markets, and infrastructure. From an economic standpoint, the most impactful factor was the total retail sales of consumer goods, exhibiting an explanatory power of 0.7672. This underscored the paramount influence of total retail sales of consumer goods on the development of the CSI in each urban district of Beijing. In line with the preceding discussion, conclusions congruent with the spatiotemporal CSI characteristics in Beijing could be drawn. In Beijing, urban areas with advanced economic development hosted a greater abundance of dining establishments, leading to a heightened concentration and diversity within the CSI. This trend was particularly evident in core and central urban areas, exemplified by districts like Dongcheng, Xicheng, and Haidian.

Population distribution influences the scale of the CSI in urban areas. In Beijing, a concentrated population positively contributed to the expansion of the urban CSI. Among the factors related to population distribution, both permanent population and the average BHI exhibited substantial explanatory power, with values of 0.6573 and 0.5877, respectively. Areas with a dense population, dynamic mobility, and robust demographic vitality tend to generate higher consumer demand, thereby fostering the development of the CSI. The population distribution in Beijing indicated a decrease in both permanent residents and the BHI from the city center toward the urban periphery. Ecological conservation areas in Beijing characterized by a relatively sparse population and limited mobility, experienced lower catering income in May and June of 2022. Conversely, core and central urban areas, featuring higher population numbers and greater demographic vitality, demonstrated elevated levels of catering income. For instance, Chaoyang District, hosting the city's largest permanent population at 3,452,460, recorded the highest catering income levels in May and June 2022. The income reached 11.3 and 18.6 billion yuan, respectively.

The convenience of service facilities plays a pivotal role in the development of CSI. Enhanced transportation accessibility benefits potential customer engagement, and dining establishments typically choose locations with convenient transportation. The clustering of various catering service facilities not only broadens service offerings and dining choices but also aids in attracting and retaining customers. Concerning service facility convenience, both transportation and catering facility densities demonstrated explanatory power, registering at 0.5877. Urban areas with more accessible transportation and a higher concentration of dining establishments tended to show increased catering income, particularly in core and central urban regions. The districts with the high service facility convenience, such as Dongcheng, Xicheng, and Chaoyang, consistently recorded elevated catering income levels.

Regarding COVID-19 risk factors, the tally of infection cases and the consecutive days with confirmed cases existing had explanatory powers of 0.5585 and 0.5902, respectively. This underscored the challenges faced by the Beijing CSI during the pandemic. With reduced population mobility due to epidemic prevention measures, there was a drastic decrease in dine-out customers. The recurrent nature of the COVID-19 pandemic disrupted traditional supply chains. While online food delivery services somewhat extended operational channels, they also increased operating costs. In an effort to avoid infection, consumers became more cautious. These factors significantly impacted the operation of catering businesses, leading many to voluntarily or involuntarily suspend operations.

First, we analyzed from the perspective of the stages of pandemic development. Official economic data for 2020 revealed substantial year-on-year revenue declined across various districts in Beijing. Regardless of the type of urban area, districts such as Haidian district experienced a 51.5% drop, while Tongzhou district showed a decrease of 43.2%. During the same period in 2021, when the pandemic was relatively stable, catering revenue across districts surged, with over 50% growth observed in many districts. However, in June 2022, data from 15 districts (excluding Yanqing district) showed a uniform decline in catering revenue, with 13 districts experiencing drops exceeding 10%.

Next, we conducted an analysis from the perspective of geographical space. During the COVID-19 outbreak in the congregated settings of the Tiantang supermarket and bars in 2022, the districts of Chaoyang, Fengtai, and Haidian, which had the highest number of COVID-19 infections, year-on-year decreases in catering revenue of 19.2%, 14%, and 15%, respectively. These declines were significant in the context of citywide catering income statistics. Dongcheng and Xicheng district were encircled by the three districts most severely affected by the pandemic. Among them, Dongcheng district's revenue fell the most at 20.7%, followed by Xicheng district at 19.1% In the previous analysis of the fluctuation in the number of catering-related POI with the severity of the pandemic, the fluctuation in the number of catering-related POI within each district was highly consistent with the catering income and the dynamics of the COVID-19 pandemic in each district. Particularly in May 2022, districts with a higher number of infections showed a noticeable reduction in the number of POI.

While the development of CSI in Beijing was influenced by multiple factors, an analysis of Beijing municipal statistics reveals a strong correlation between the positive or negative growth of catering income in various districts and the pandemic situation. During large-scale clustered outbreaks of COVID-19, catering revenue in Beijing decreased year-on-year, particularly in districts heavily affected by the pandemic, resulting in significant negative growth rates. Conversely, when the pandemic situation was stable, catering revenue across districts exhibited a year-on-year positive growth trend. It can be inferred that the outbreak of congregated COVID-19 cases has a detrimental effect on catering income in the affected areas, and the dynamics of the COVID-19 pandemic directly impact the development of the CSI in these regions.

## Discussions

In this study, we thoroughly examined the dynamic evolution of the spatial distribution of the Beijing CSI during the COVID-19 pandemic. This investigation uncovered the heterogeneity in the spatiotemporal patterns of the Beijing CSI. While some scholars analyzed the spatiotemporal characteristics of CSI during the pandemic and explored its impacts using multiple data sources, most studies focused on the overall spatial characteristics of the CSI at a single time point. In contrast, our approach employed POI data from multiple time points, covering a span of six years from 2018 to 2023. This allowed for a relatively long-term monitoring of CSI changes and development. The temporal granularity of our data analysis was specified down to individual months, facilitating a close alignment with key developments in the COVID-19 pandemic. Based on the *Beijing Urban Master Plan*, the city was divided into four urban categories: the core urban areas, central urban areas, peripheral urban areas, and ecological conservation areas. This classification facilitated a more in-depth analysis of the temporal and spatial variations in the distribution characteristics of the CSI. The study not only scrutinized the overall spatiotemporal patterns of the CSI but also refined the categorization of catering service facilities. The inclusion of spatiotemporal characteristics from different types of catering spaces enriched the diversity of our analysis. Finally, this paper explored the factors that influenced the development of the CSI in Beijing. The research also considered the interactions and impacts among various types of living service sectors. From diverse perspectives, including the social, economic, and the repercussions of the COVID-19 pandemic, the paper examined the development of the CSI under the collective influence of multiple factors.

The overall distribution of Beijing CSI showed a gradual decline from the city center to the periphery. In the urban center, there were more catering service facilities, higher concentration, and a greater variety of offerings, resulting in increased catering revenue. Moving towards the urban periphery, especially in the ecological conservation areas, catering service facilities were more sparsely distributed, with lower functional diversity, leading to reduced catering income in these regions. However, during periods of the recurrent COVID-19 pandemic, the spatial characteristics of the CSI varied across different years. In times of more severe COVID-19 outbreaks, the development of Beijing CSI faced limitations. During such periods, the spatial features of Beijing CSI exhibited more pronounced differences compared to periods when the pandemic situation was relatively stable. For instance, there were fewer catering service facilities, lower concentration, and reduced diversity during periods of heightened pandemic impact.

The heterogeneity of this spatiotemporal pattern was influenced by multiple factors. The central region of Beijing, characterized by higher economic levels, a more concentrated population, and greater convenience of service facilities, contributed to the overall spatial pattern of Beijing's CSI. According to the *China Catering Industry Development Report*, during the COVID-19 epidemic, catering enterprises needed to bear the rising costs of raw materials, labor, rent and other costs. Additionally, catering enterprises took on social responsibility and increased multiple epidemic prevention expenses. The reduction in foot traffic and a decline in consumer willingness to dine out made the operation of catering businesses more challenging. Nevertheless, the CSI in the central of Beijing demonstrated greater resilience under the impact of the pandemic, aligning with the characteristics of the spatial pattern mentioned above.

## Conclusions

This paper was based on catering-related POI data from different times spanning 2018 to 2023 in Beijing. We combined multiple data sources and employed methods including KDE, SDE, SHDI, correlation analysis, and Geographic detectors. The time granularity of the big data analysis was precisely measured down to a specific year and month. Focusing on critical phases of the pandemic's development, it extensively analyzed the spatiotemporal heterogeneity and driving factors of Beijing's CSI. The following conclusions are drawn:

(1) Temporal trends in the distribution of Beijing's CSI were examined. The research findings revealed that the spatiotemporal characteristics of the CSI in Beijing exhibited a "W" shaped fluctuation trend from 2018 to 2023. The dynamic changes in the quantity, concentration, and functional diversity of catering establishments were closely associated with the evolving pandemic conditions. Turning points were notably observed around critical phases in the progression of the COVID-19 pandemic, specifically in November 2020, December 2021, and May 2022. During periods of heightened COVID-19 outbreaks, Beijing's CSI exhibited a decrease in the number of catering establishments, a reduction in concentration, and a decline in diversity. (2) The geographical distribution characteristics of CSI in Beijing were examined. Against the backdrop of the COVID-19 pandemic, the spatial characteristics of Beijing's CSI displayed a strengthening "center-to-edge" pattern of changes. The stability of CSI in the city center was more pronounced, while the peripheral areas experienced higher variability. Meanwhile, regions with more complex epidemics experienced more significant fluctuations in catering revenue. (3) Changes in various catering spaces. Chinese restaurants showed the least variation, indicating higher resilience and stability. In contrast, fast food restaurants, beverage and dessert shops exhibited lower stress resistance but a greater capacity for recovery. (4) The development of CSI in Beijing was influenced by multiple factors. Various categories within the lifestyle service industry in Beijing exerted mutual influence and constraints. Positive impetuses for CSI development were found in the economic level, population distribution, and accessibility of service facilities. Conversely, the development of CSI was hindered by the COVID-19 pandemic.

The COVID-19 pandemic no longer constitutes an "internationally significant public health emergency." Although new variants of the coronavirus are still under surveillance, the World Health Organization has reported a gradual decline in confirmed cases and deaths related to COVID-19. People's lives have returned to normal, and various industries are experiencing rapid growth. This paper conducted a detailed analysis of the spatiotemporal heterogeneity of CSI during the COVID-19 pandemic in Beijing. This analysis aimed to provide insights for the development of the CSI and other sectors in the future, even in the event of similar emergencies, serving as a reference for government decision-making.

Undoubtedly, this study has its limitations. Beyond the need for methodological enhancements, the accuracy of multi-source big data, including POI, requires further scrutiny. As online mapping technologies advance and data collection methods mature, we aim to find more effective solutions to address the inherent 'lag' issue in POI data. Concurrently, we are confident that integrating innovative and diverse analytical methods with data sources like POI will become even more seamless. Multi-source big data, including POI, is poised to deliver intelligent services across various applications for both government and the public.

## Data Availability

The datasets used and/or analysed during the current study available from the corresponding author on reasonable request.
